# Control of Rubisco function via homeostatic equilibration of CO_2_ supply

**DOI:** 10.3389/fpls.2015.00106

**Published:** 2015-02-26

**Authors:** Abir U. Igamberdiev

**Affiliations:** Department of Biology, Memorial University of Newfoundland, St. John’s, NL, Canada

**Keywords:** bicarbonate pool, carbonic anhydrases, chloroplasts, Michaelis–Menten kinetics, photorespiration, reactant stationary approximation, Rubisco

## Abstract

Rubisco is the most abundant protein on Earth that serves as the primary engine of carbon assimilation. It is characterized by a slow rate and low specificity for CO_2_ leading to photorespiration. We analyze here the challenges of operation of this enzyme as the main carbon fixation engine. The high concentration of Rubisco exceeds that of its substrate CO_2_ by 2–3 orders of magnitude; however, the total pool of available carbon in chloroplast, i.e., mainly bicarbonate, is comparable to the concentration of Rubisco active sites. This makes the reactant stationary assumption (RSA), which is essential as a condition of satisfying the Michaelis–Menten (MM) kinetics, valid if we assume that the delivery of CO_2_ from this pool is not limiting. The RSA is supported by active carbonic anhydrases (CA) that quickly equilibrate bicarbonate and CO_2_ pools and supply CO_2_ to Rubisco. While the operation of stromal CA is independent of light reactions, the thylakoidal CA associated with PSII and pumping CO_2_ from the thylakoid lumen is coordinated with the rate of electron transport, water splitting and proton gradient across the thylakoid membrane. At high CO_2_ concentrations, CA becomes less efficient (the equilibrium becomes unfavorable), so a deviation from the MM kinetics is observed, consistent with Rubisco reaching its *V*max at approximately 50% lower level than expected from the classical MM curve. Previously, this deviation was controversially explained by the limitation of RuBP regeneration. At low ambient CO_2_ and correspondingly limited capacity of the bicarbonate pool, its depletion at Rubisco sites is relieved in that the enzyme utilizes O_2_ instead of CO_2_, i.e., by photorespiration. In this process, CO_2_ is supplied back to Rubisco, and the chloroplastic redox state and energy level are maintained. It is concluded that the optimal performance of photosynthesis is achieved via the provision of continuous CO_2_ supply to Rubisco by carbonic anhydrases and photorespiration.

## INTRODUCTION

Carbon fixation can be achieved by chemically simple processes, and it is enigmatic that it occurs via the complex mechanism mediated by a slow enzyme with dual specificity. Rubisco serves as the primary engine of carbon assimilation being the most abundant protein on Earth and characterized by a slow rate of catalysis (in higher plants the value of *k* is approx. 3 s^-1^) and low specificity for CO_2_ leading to photorespiration. As a simple alternative, the reduction of CO_2_ to formate which is then metabolized to carbohydrates and other compounds (C_1_-pathway of carbon assimilation) is possible. This mechanism, with no connection to the reductive photosynthetic carbon pathway (Calvin–Benson cycle), exists in some prokaryotes (Tagawa and Arnon, 1962). The reaction of carbohydrate synthesis from one-carbon compounds was first demonstrated in organic chemistry by Butlerow (1861); then Baeyer (1870) suggested formaldehyde as the primary product of CO_2_ fixation by plants. This hypothesis dominated before the discovery of the Calvin–Benson cycle in the 1940s (Benson and Calvin, 1950). Whereas it is now widely accepted that formate is not involved in the primary CO_2_ assimilation processes in plants, it may still have a role in side-reactions of photosynthesis and photorespiration, also including a possibility of a very minor CO_2_ fixation pathway via formate and formaldehyde (Kent, 1972; reviewed in Igamberdiev et al., 1999). However, this simple pathway did not acquire major significance during evolution and instead the enzyme Rubisco became the primary engine of carbon assimilation. In this paper, we demonstrate that by using Rubisco, which seems to be inefficient due to low turnover number and low specificity, living systems in fact exploit the mechanism that provides the most optimal parameters of carbon fixation. This is realized by coordinating CO_2_ assimilation with generation of reducing power and energy currency and with oxygen utilization.

## CONDITIONS FOR MICHAELIS–MENTEN KINETICS

It is widely assumed that all enzymes follow the Michaelis–Menten (MM) kinetics. The derivation of MM equation is usually based on the steady-state assumption (SSA) that the concentration of substrate (S) should be much higher than the concentration of enzyme (E), so practically all the enzyme during the reaction exists in the state of enzyme-substrate complex (ES). Recently it was shown that the SSA condition is not crucial, while essential is the reactant stationary assumption (RSA). It means that there is an initial transient phase, during which the initial substrate concentration remains approximately constant, while the ES complex concentration builds up (Schnell, 2014). This truly necessary condition does not require the restrictive limitation of choosing a substrate concentration that is much higher than the enzyme concentration in initial rate experiments. For the RSA to be valid, there must be only a negligible decrease in S during the initial transient phase. An auxiliary enzyme that supplies substrate from the reserve pool can provide this condition. This enzyme (called the *buffering enzyme*) should be fast as compared to the enzyme present in high concentration (called the *engine enzyme*; Igamberdiev and Kleczkowski, 2009). The combination of the fast (buffering) enzyme catalyzing the equilibrium reaction and the slow (engine) enzyme catalyzing the non-equilibrium reaction ensures that the total reaction rate is optimized via constant building up of the ES complex of the engine enzyme. This condition can be defined as the stable non-equilibrium principle following Bauer (1935).

Even at lower concentrations of substrate, the MM kinetics may be valid if the substrate is constantly delivered from its major reserve pool. The simplest way of such delivery occurs via a buffering enzyme. This was demonstrated earlier for ATP synthase, when its substrate (ADP) is delivered by the buffering enzyme adenylate kinase (Igamberdiev and Kleczkowski, 2003, 2006, 2015). Another example is glycine decarboxylase, when the buffering of its end products, NADH and CO_2_, by malate dehydrogenase and carbonic anhydrase (CA), respectively, is important for maintaining its maximal activity (Bykova et al., 2014). The same role of malate dehydrogenase has been established for the mitochondrial pyruvate dehydrogenase complex, which in plants has also the mechanism of substrate delivery via NAD-malic enzyme (Igamberdiev et al., 2014). For Rubisco, the condition of optimization of CO_2_ supply was outlined previously (Igamberdiev and Roussel, 2012), where it was assumed that neither SSA nor RSA conditions could be satisfied due to a very low CO_2_ concentration in chloroplast stroma as compared to Rubisco concentration.

**FIGURE 1:**
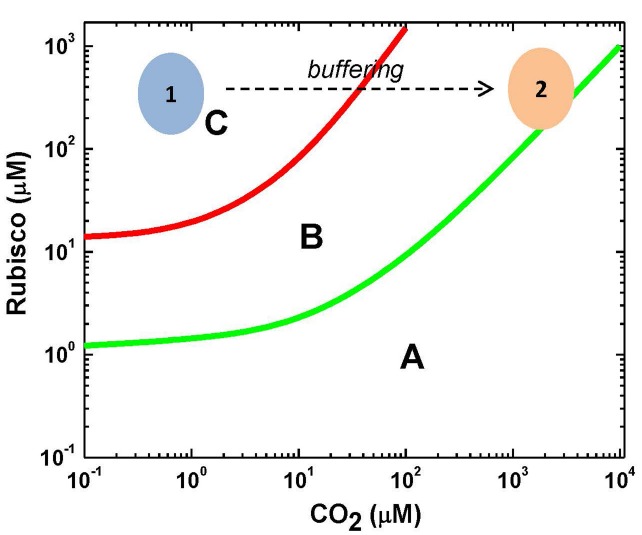
**Limits of validity of SSA and RSA for the Rubisco reaction based on the approach developed in Hanson and Schnell (2008).** Area **(A)**: both the RSA and SSA are valid and the reaction obeys the MM equation. Area **(B)**: the SSA is valid but the RSA is not. Applying the MM equation is possible provided that the decrease in substrate concentration is negligible during the initial transition. This can be achieved by thermodynamic buffering. Area **(C)**: neither the QSSA nor the RSA is valid, substrate is rapidly depleted by enzyme and its continuous influx is necessary for steady reaction. The area 1 marks the *in vivo* condition if we assume that only CO_2_ is used as a substrate, while the area 2 marks the *in vivo* condition when we assume that the whole bicarbonate/CO_2_ pool is used as a substrate, provided that CO_2_ is efficiently delivered by the buffering enzyme carbonic anhydrase.

In the current paper, it is suggested that the situation is not so far-reaching due to a high CA capacity making the MM kinetics generally valid for Rubisco, while the restrictions appear at low CO_2_ (the oxygenase reaction present) and high CO_2_ (CA equilibrium is less favorable) concentrations. The whole bicarbonate pool of chloroplast can be considered as a substrate for Rubisco, provided that CO_2_ is efficiently delivered to its active sites. This is reflected in Figure 1, which depicts the conditions of SSA, RSA, and MM, depending on CO_2_ and Rubisco concentrations. It shows also actual concentration values for Rubisco and CO_2_ *in planta* if we consider either only the dissolved molecular CO_2_ or the CO_2_ that can be delivered from the bicarbonate reserve pool. The kinetic analysis suggests that in the region where the RSA is valid but the SSA is not, the estimates of *V*_max_ and *K*_m_ are highly inflated (Hanson and Schnell, 2008). The increase in *V*_max_ indicates that the rate of reaction can be increased significantly at the expense of the affinity to substrate; however, the conditions of the steady delivery of substrate by the buffering enzyme successfully overcome this limitation.

## CARBONIC ANHYDRASE AND CO_2_ DELIVERY CAPACITY

The buffering of energy intermediates is the most widespread energy source in biological systems, and filling buffer reservoirs corresponds to the accumulation of free energy (Shnoll, 1979). Within the framework of the theory of dissipative structures (Prigogine, 1967), thermodynamic buffering (Stucki, 1980a) represents the basic regulatory principle for the maintenance of a stable far-from-equilibrium regime, where the production of energy is minimal. The bicarbonate buffering via CA operates effectively at pH values close to neutral and plays a significant role in many physiological processes—from carbon fixation in photosynthesis to respiration in animals (Henry, 1996). In the Rubisco reaction, CA will provide a continuous delivery of CO_2_ from the bicarbonate pool.

According to the Henderson–Hasselbalch equation, only 5–7% of CO_2_ is released from bicarbonate and carbonate at physiological pH (more with pH decrease). A facilitation of the equilibrium reaction between CO_2_ and bicarbonate by CA will promote a supply of CO_2_ to Rubisco. The large pH gradient between the cytosol and chloroplast stroma (which becomes higher in the light) results in a HCO_3_^–^ concentration that is about five times higher in the stroma compared with the cytosol (Werdan and Heldt, 1972). The transition from darkness to light increases the HCO_3_^–^ concentration of chloroplast stroma from 0.5 to 2–2.5 mM (Sicher, 1984). This makes the total concentration of carbon accessible to Rubisco approximately equal to the concentration of Rubisco active sites, resulting in the conditions where RSA is valid, provided that the delivery of CO_2_ from this pool is not limited. However, the data obtained with the stromal CA mutant indicate that the presence of stromal CA does not provide very significant facilitation of CO_2_ delivery from the stromal pool (Price et al., 1994). On the other hand, the reduction in chloroplast CA affects survival of plants at earlier stages of development (Ferreira et al., 2008). Also, the suppression of CA strongly affects photosynthetic rate of CO_2_ assimilation (Igamberdiev and Roussel, 2012). The importance of CA in the optimal Rubisco performance is also shown for cyanobacteria (Nishimura et al., 2014) and in photosynthetic bacteria (Dou et al., 2008), but in these organisms it can be explained by the existence of CA-based carbon concentration mechanism. A possible contribution of different CA isoforms to CO_2_ fixation by higher plants will be discussed below.

Evans (2009) estimated that, in the conditions of fully established CA equilibrium, the CO_2_ flux in the chloroplast stroma will increase by 26 times as compared if CO_2_ were the only diffusing substance. According to Tholen and Zhu (2011), the complete equilibration of CO_2_ and HCO_3_^–^ occurs at CA concentration of 1 mM, while the concentration of CA in chloroplast stroma ranges between 0.04 and 0.69 mM, suggesting that the amount of CA may somewhat limit the conductance in the stroma. According to their estimations, the internal conductance of CO_2_ would decrease by more than 40% without CA, decreasing photosynthesis only by 7%. This small value, however, does not include the contribution of the thylakoidal CA that can supply CO_2_ to Rubisco depending on the electron transport rate (Shutova et al., 2008). This CA isoform was discovered in algae where it is considered to be most important for feeding CO_2_ to Rubisco (Hanson et al., 2003); recent studies indicate that it is present also in higher plants (Ignatova et al., 2011). This CA is also important for PSII operation because, simultaneously with CO_2_ production, it removes proton from the thylakoid lumen relieving inhibition of photosynthetic electron transport (Shutova et al., 2008). The primary contribution of the thylakoidal CA to CO_2_ delivery to Rubisco can explain why the rate of photosynthesis in the stromal CA mutant was not very significantly affected (Price et al., 1994). This explains also why the transgenic plants with impaired stromal CA provided no conclusive evidence for the important role of CA in photosynthesis. However, the fast delivery of CO_2_ at its higher concentration may be achieved mainly via the stromal CA operating at pH ∼8, because the CA equilibrium is less favorable in the thylakoid lumen at low pH values and not sufficient to feed Rubisco. Up to four separate CA activities associated with thylakoids have been identified (Rudenko et al., 2007).

**FIGURE 2:**
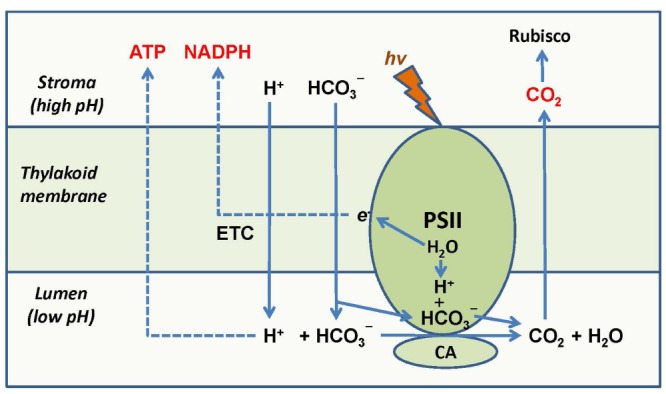
**Coordination of supply of NADPH, ATP, and CO_2_ to Rubisco by PSII and its CA-associated activity.** PSII supplies electrons to the chloroplast electron transport chain which results in NADP^+^ reduction and generation of proton gradient. The protons released during water splitting are accepted by bicarbonate anions forming CO_2_. A part of the proton gradient is used for bicarbonate transport in the lumen where it forms CO_2_ with the help of the PSII-associated CA. CO_2_ is thus supplied to the stroma and feeds Rubisco. Modified from Igamberdiev and Roussel (2012).

The involvement of the thylakoidal CA in CO_2_ delivery to Rubisco implies that the operation of chloroplast electron transport not only supplies NADPH and ATP to the Calvin–Benson cycle, but also participates in pumping CO_2_ to Rubisco in the stroma through the CA mechanism. This mechanism is linked to the CA activity of PSII and correlates the intensity of chloroplast electron transport and photophosphorylation with the intensity of the Calvin–Benson cycle. Bicarbonate influx to the thylakoid, the CO_2_ supply to Rubisco, water splitting, and the build-up of proton gradient are all coupled via the CA activity of PSII (Figure 2). Recent findings show that bicarbonate acts as proton acceptor in photosynthetic water oxidation (Koroidov et al., 2014) and that the efficiency of water oxidation depends on the levels of inorganic carbon (Shevela et al., 2013). Bicarbonate is involved in transport of protons produced by water oxidation inside of photosystem II out into the lumen, resulting in a light-driven production of O_2_ and CO_2_ that can be delivered to Rubisco. The depletion of bicarbonate leads to a reversible down-regulation of O_2_ production (Koroidov et al., 2014). The dependence of PSII activity on HCO_3_^–^ concentration also allows for sensing inorganic carbon level in the stroma and coordinating a feedback regulation of PSII with the Calvin–Benson cycle. These findings add bicarbonate to the regulatory network of oxygenic photosynthesis and confirm that CO_2_ is released in concordance with the operation of PSII, and that PSII together with the associated CA directly participates in CO_2_ production from bicarbonate.

The thylakoidal CA contributes to the equilibrium of bicarbonate and CO_2_ and thus to the conversion of the bicarbonate pumped inside the thylakoid (by using the proton gradient formed during operation of the photosynthetic electron transport chain) to CO_2_ which escapes back to the stroma. This concentration mechanism was initially suggested by Fridlyand (1995). As it was mentioned earlier (Igamberdiev and Roussel, 2012), at high CO_2_ concentrations this mechanism will have a higher energy expenditure (dissipation of proton gradient) which may lead to a depletion of ATP needed for regeneration of the CO_2_ acceptor in the Calvin–Benson cycle. It thus explains the observed decrease in the rate of photosynthesis at oversaturating CO_2_ concentrations, e.g., in wheat plants (Fridlyand and Tsyuryupa, 1992) and in pea protoplasts (Riazunnisa et al., 2006). As the PSII-driven electron transport is a feedforward regulator of CO_2_ assimilation, the PSII-associated CA greatly amplifies the flux of CO_2_ available for carboxylation (Park et al., 1999). The stromal CA can also participate in supplying CO_2_ to Rubisco but it apparently lacks the necessary efficiency in CO_2_ concentration, therefore the thylakoidal CA can be considered as the main mechanism providing the continuous CO_2_ delivery to Rubisco.

Hanson et al. (2003) have shown that insufficient activity of the thylakoidal CA leads to a strong decrease in CO_2_ fixation. This can be compared with a much weaker effect on photosynthesis by decreasing stromal CA activity (Tholen and Zhu, 2011). The thylakoidal CA operates at low pH where higher portion of CO_2_ is present as a dissolved gas or H_2_CO_3_. This means that its efficiency in CO_2_ delivery may decrease already with a moderate increase of CO_2_ supply. The depletion of CO_2_ substrate at Rubisco site is similar to what Chance and Williams (1955) described for ATP synthase by introducing the term “State 4” to characterize the steady state with a vanishing flow ratio. In the case of the mitochondrial ATP synthase, State 4 condition is avoided *in vivo* due to the action of adenylate kinase equilibrating adenylates in the mitochondrial intermembrane space (Igamberdiev and Kleczkowski, 2003, 2006, 2015). The depletion of CO_2_ at the active sites of Rubisco is similar to State 4, and it is avoided by the buffering role of CA. This means that CA works as a thermodynamic buffer enzyme, which is able to effectively buffer the carboxylation potential to the value permitting optimal efficiency of Rubisco reaction. In fact, Rubisco is well optimized to changing CO_2_, O_2_, and thermal conditions in the subcellular environments (Tcherkez et al., 2006). The increased efficiency of photosynthesis can be likely achieved not through modulation of the Rubisco enzyme itself but rather via engineering of bicarbonate pumps and increasing their efficient coupling with the Rubisco reaction, e.g., by using transgenic plants expressing the genes associated with cyanobacterial carbon concentration mechanisms (Price et al., 2013; Zarzycki et al., 2013; McGrath and Long, 2014).

## RUBISCO CURVE AND ITS INTERPRETATION

The dependence of Rubisco activity on CO_2_ in tissues and isolated chloroplasts is represented by a characteristic kinetic curve, in which the deviation from hyperbolic kinetics takes place upon the increase of CO_2_ concentration. Laisk (1985), Laisk and Oja (1998), and Ruuska et al. (1998) demonstrated a deviation from the MM curve by reaching a maximum velocity of *V*_max_ that ranges from 25 to 50% of the transitional maximum velocity (*V*_Cmax_) that could be achieved in the absence of such deviation. This means that the rate of steady state reaction deviates from that of the transitional state before CO_2_ concentration reaches its constant level defined in frames of the MM kinetics. In order to explain the unusual shape of Rubisco curve, it has been suggested that the limitation comes from regeneration of RuBP (Laisk, 1985; Laisk and Oja, 1998; Laisk et al., 2009). This explanation was questioned by Farazdaghi (2011): according to his views, the whole RuBP regeneration process never affects directly the steady state rate of Rubisco reaction but instead the inhibition of Rubisco by the immediate product of reaction (PGA) is involved. The main problem in this interpretation is the lack of experimental data supporting such inhibitory mechanism.

The dependence of *k*_cat_ of Rubisco *in planta* (as measured in intact leaves) on photosynthetic electron transport (Eichelmann et al., 2009) is firmly established; however, the final explanation is not given. There is no direct evidence that it occurs via PGA accumulation. Since the rate of CO_2_ supply via the thylakoidal CA associated with PSII is directly dependent on electron transport, its intensity can determine the maximum rate of Rubisco, which can vary by one order of magnitude depending on the electron transport rate (Eichelmann et al., 2009). It is shown that the thylakoid lumen-localized CA limits CO_2_ supply to Rubisco, while the pool of RuBP is not depleted (Hanson et al., 2003). The limitation of CO_2_ supply from thylakoid can occur not only due to its dependence on electron transport but also because, at high CO_2_, the CA equilibrium becomes less favorable, whereas the CO_2_ concentration is still far below the concentration of Rubisco active sites. The mechanism of CO_2_ supply would prevent the overproduction of PGA above the capacity of its utilization in the Calvin–Benson cycle; moreover at high CO_2_ its production may be kept below such capacity due to earlier saturation of Rubisco curve, which becomes the cause rather than the consequence of the observed shape of Rubisco curve.

The involvement of buffering activities for removal of product of enzyme complexes has been shown for glycine decarboxylase complex (Bykova et al., 2014) and for pyruvate dehydrogenase complex (Igamberdiev et al., 2014). In both cases, the buffering enzymes establish rapid equilibrium of the products of enzymatic reaction relieving inhibition by the product at the active site. For these two enzymes, malate dehydrogenase establishes NADH/NAD^+^ equilibrium, favoring low concentration of NADH that is a strong inhibitor of both enzymes. In the case of glycine decarboxylase, CA may be another important buffering enzyme, converting the formed in the reaction CO_2_ to bicarbonate. In the case of Rubisco, the suggested limitation by the product PGA (Farazdaghi, 2011) is less evident, although the antisense reduction of glyceraldehyde phosphate dehydrogenase activity also limits the flux through Rubisco (Price et al., 1995), and the reaction of PGA conversion to glyceraldehyde-3-phosphate is displaced strongly toward the product formation, depending on ATP/ADP ratio in the stroma (Fridlyand and Scheibe, 1999). More important is the equilibration of glyceraldehyde-3-phosphate and subsequent substrates in further reactions equilibrated by buffering enzymes of the Calvin–Benson cycle (Igamberdiev and Kleczkowski, 2011). Thus, without the denial of possible Rubisco limitation by PGA, we are more in favor of limitation by CO_2_, which can take place both at the level of Rubisco activation by CO_2_ binding and at the level of Rubisco catalysis.

Considering the source of CO_2_ as bicarbonate buffer, we can, following (Stucki, 1980a,b) and his theory of thermodynamic buffering (reviewed in Igamberdiev and Kleczkowski, 2009), instead of the dependence of maximum velocity from substrate concentration, consider the dependence of flow ratio *j* on force ratio *x* (Stucki, 1980a):

j=(x+q)/(qx+1)

This dependence will be expressed by the degree of coupling *q*, the parameter which was considered by Stucki (1980a) as constant. However, in the case of Rubisco it will have higher value at low CO_2_ concentration due to CA equilibrium strongly displaced toward CO_2_, while at high CO_2_ concentration its value will be lower due to less favorable equilibrium of CA for CO_2_ supply to Rubisco. This means that at the force ratio approaching zero (the change from transitional to steady state) the flow ratio will be lower than that achieved at higher degree of coupling that occurs in the transitional state. This means that the dependence of flow through Rubisco (assimilation of CO_2_) will follow the hyperbolic kinetics at low CO_2_ concentration (low force of CO_2_ supply to Rubisco with high coupling) which then will be saturated at significantly lower rate than that following from MM kinetics due to lower coupling at the high force of CO_2_ supply.

**FIGURE 3:**
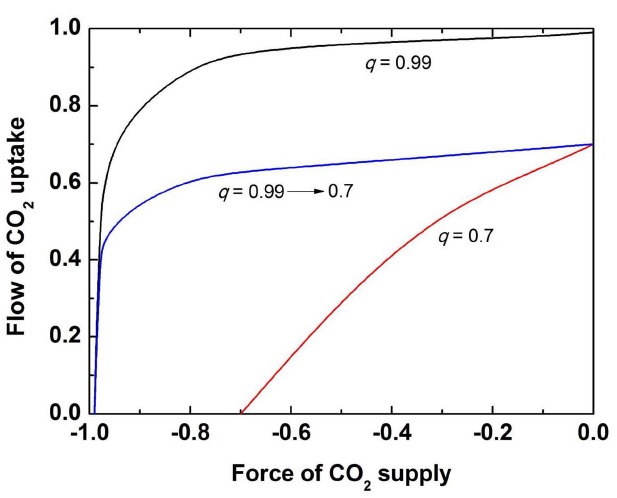
**Dependence of the flow rate (utilization of CO_2_ by Rubisco) on force rate (the level of saturation by CO_2_) at different values of the coupling parameter *q* as indicated in the figure (high degree of coupling, *q* = 0.99; low degree of coupling, *q* = 0.7; the decreasing degree of coupling from 0.99 to 0.7 which approximates to the actual Rubisco curve)**.

In the conclusion of this section, it should be stated that the Rubisco curve is not a classical MM curve; however, it coincides with the MM curve when the delivery of CO_2_ from the CO_2_/bicarbonate pool is not limited by the buffering enzyme (CA). The limitation comes when CO_2_ concentration becomes high and CA equilibrium turns to be less efficient to produce CO_2_ from bicarbonate. The dependence of Rubisco reaction from CO_2_ is the dependence of flow ratio (Rubisco reaction) on force ratio (delivery of CO_2_). While at low CO_2_ concentration the coupling coefficient *q* of CA approaches almost 1, upon CO_2_ increase CA becomes less efficient and the coupling between CO_2_ and its delivery to Rubisco drops down. Figure 3 shows the dependence of flow ratio on force ratio at two different degrees of coupling (0.99 and 0.7) and when the coupling coefficient continuously decreases from 0.99 to 0.7. As a result, we obtain a curve which is saturated at lower level of flow rate than it is expected from the MM equation, as observed in the actual case of Rubisco *in planta*. This shape of curve simply means that the lower value of saturating Rubisco velocity than the *V*_max_ value expected from the MM kinetics occurs due to a decreasing capacity of CO_2_ supply by CA at increased CO_2_ concentrations. The mechanism of CO_2_ supply by CA that pumps it from the thylakoid lumen can efficiently deliver CO_2_ to Rubisco, but its efficiency decreases with the increase of CO_2_ concentration.

One of the important features of Rubisco is its carbon isotope effect. ^13^C carbon isotope discrimination values are stable for concrete Rubiscos and differ depending on Rubisco type and evolutionary position of organism (von Caemmerer et al., 2014). According to the classical theory, the Rayleigh effect (Rayleigh, 1896) should take place, affecting the discrimination value at low CO_2_. The significant constancy of Rubisco carbon isotope effect may indirectly indicate that CO_2_ at Rubisco sites is never depleted because it is delivered by the buffering enzyme CA. At the rate of delivery determined only by the rapid equilibrium achieved via CA, the constant ^13^C fractionation values will represent the *in vivo* property of Rubisco under the continuous flux of CO_2_. Variation in ^13^C discrimination depending on CO_2_ depletion may take place in the conditions of low CA activity or its inhibition, e.g., it was shown that carbon isotope fractionation by Rubisco is lower if the stromal CA is reduced by 92% (Williams et al., 1996), confirming that CA indeed prevents CO_2_ depletion at Rubisco active sites.

## THE ROLE OF PHOTORESPIRATION IN CO_2_ SUPPLY TO RUBISCO

While the mechanism of control of Rubisco function via CO_2_ buffering by CA turns the whole bicarbonate pool to a substrate with high degree of coupling, at low ambient CO_2_ the bicarbonate/CO_2_ pool in C_3_ plants is not renewed sufficiently fast upon depletion. In those conditions, Rubisco load by the substrate remains insufficient to provide the turnover of the Calvin–Benson cycle to consume the reductive power and energy from light reactions. A lower activation state of Rubisco at low CO_2_ can partially contribute to the overall balance of redox and energy but the most important tool in these conditions becomes the second mechanism of Rubisco optimization, which is its oxygenase reaction. At low CO_2_ concentrations, even at high degree of CA coupling, the flow through Rubisco is low, since CO_2_ delivery remains restricted even at the high degree of coupling between CA and Rubisco leading to a decrease of the reaction rate. This results in displacement of the balance between the electron transport rate and CO_2_ fixation. The CO_2_ load of carbon assimilation that is attributed to carboxylation at Rubisco site is not constant but fluctuates depending on CO_2_ supply. Therefore a device is sought which can compensate the fluctuations of the load in order to minimize the deviations from conductance matching and hence the deviations from optimal efficiency of carboxylation *in vivo*.

In the conditions of low ambient CO_2_, the oxygenase reaction of Rubisco is needed to compensate fluctuations of the load. Depending of the ambient O_2_ and CO_2_ concentrations, the Rubisco kinetics is described by the two mirror curves producing the same optimal flow, i.e., the flux via activated Rubisco is apparently constant at different CO_2_ concentrations (André, 2011). The oxygenase reaction of Rubisco takes an alternative substrate (O_2_) and provides the CO_2_ production in the photorespiratory sequence of reactions. This restricts the variation of CO_2_ and O_2_ concentrations within certain limits and keeps the flux through Rubisco constant (Roussel and Igamberdiev, 2011). At low CO_2_, the supply of CO_2_ to Rubisco becomes uncoupled from the activity of PSII and the generation of NADPH and ATP exceeds the capacity of the Calvin–Benson cycle. The use of oxygen keeps the flux through Rubisco steady by initiating a metabolic pathway that serves as a major sink of reducing power and ATP.

An increase in photorespiration reduces net photosynthesis but contributes to the maintenance of chloroplast CO_2_ concentration (Tholen and Zhu, 2011). The mechanism of delivery of substrate from the products of reaction is well explored in nature. The pair of flavin-containing oxidase and catalase in peroxisomes represents such example (Igamberdiev and Kleczkowski, 2009). By returning half of the oxygen consumed in the flavin-dependent reaction, catalase not only detoxifies hydrogen peroxide but also prevents depletion of oxygen at the site of flavin-containing oxidase. Photorespiration produces CO_2_ in the quantity of 25% of the phosphoglycolate carbon. Oxidation of glyoxylate by the flavin-dependent glycolate oxidase is fast and non-limited by redox level, as this could be in the case of glycolate dehydrogenase (Igamberdiev and Kleczkowski, 2009). The mitochondrial form of CA may contribute to efficient reutilization of the photorespiratory CO_2_ (Bykova et al., 2014). The intercompartmental pattern of the photorespiratory pathway promotes metabolite channeling, contributes to the control of carbon flux routes through the metabolic network (Sweetlove and Fernie, 2013) and generates the oscillatory regime between the reactions of carboxylation and oxygenation (Roussel et al., 2007; Roussel and Igamberdiev, 2011).

Thus, the role of photorespiration consists not only in the maintenance of redox and energy balance of photosynthetic cells of C_3_ plants by utilization of the excess of NADPH and ATP, but also in preventing CO_2_ depletion at Rubisco sites. Photorespiration leads to the maintenance of the balance between CO_2_ load and consumption of NADPH and ATP, so the processes become well equilibrated. In C_4_ plants, which remain phenotypically less plastic (Sage and McKown, 2006), the role of photorespiration becomes minimal; however, it may still play a role in bundle sheath cells (Yoshimura et al., 2004) where all photorespiratory CO_2_ is efficiently captured. Photorespiration may also be a factor that prevented depletion of CO_2_ concentration in the atmosphere (Igamberdiev and Lea, 2006) in the same way as it prevents its depletion in photosynthetic cells. Engineering of plants with a high capacity of bicarbonate pump can also result in more efficient refixation of photorespiratory CO_2_; however, modulation of the photorespiratory pathway itself could lead to the changes in the coordination between Rubisco activity, electron transport rate and cellular redox balance, and therefore may not be beneficial for the overall photosynthetic performance (de Carvalho et al., 2011).

## CONCLUSION

**FIGURE 4:**
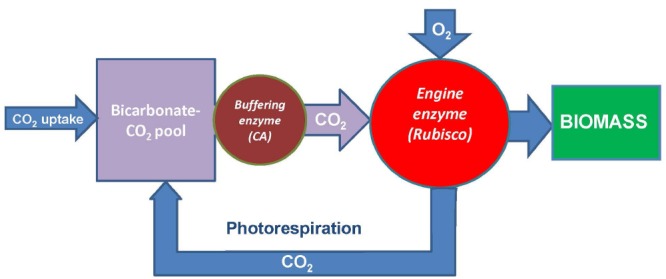
**General scheme showing operation of Rubisco as the engine for generating biomass from CO_2_. The source of CO_2_ is a** bicarbonate pool fed from the atmosphere and buffered by carbonic anhydrase (CA) serving as a pump for Rubisco. The latter is an engine producing biomass and at the same time generating a feedback (photorespiration) via its oxygenase reaction to feed the bicarbonate pool in the conditions of insufficient CO_2_ supply. Modified from Igamberdiev and Roussel (2012).

Rubisco operates at much higher concentration than its substrate CO_2_; however, chloroplasts have bicarbonate pool which concentration is comparable to the concentration of Rubisco and increases upon illumination. The delivery of CO_2_ from this pool to Rubisco active sites is a major prerequisite of its stable operation. While the stromal CA has a limited capacity for provision of the optimal performance of Rubisco, the mechanism involving the PSII-associated CA activity coordinated with water splitting, chloroplast electron transport, and ATP synthesis may possess a higher capacity. At low ambient CO_2_, this mechanism cannot produce enough CO_2_ to utilize all the reducing power generated in light reactions, and the oxygenase reaction of Rubisco represents a sink for reducing power and energy and results in photorespiratory supply of CO_2_ to keep Rubisco functioning. The flux through the activated Rubisco is apparently steady and relatively independent of different CO_2_ concentrations (considering that CO_2_ can be substituted by O_2_). At low CO_2_, its supply is limited by the photorespiratory feedback, while at high CO_2_ it is limited by the capacity of the thylakoidal CO_2_ pump. This is summarized in Figure 4. Rubisco operates at lower CO_2_ concentrations than its protein concentration, while the homeostatic equilibration of CO_2_ supply from the bicarbonate pool controls the metabolic flux through the enzyme and coordinates it with NADPH and ATP produced in light reactions. By using Rubisco, which originally seemed to be inefficient having low catalytic constant and low specificity, living systems in fact exploit the mechanism that aims to achieve the optimal parameters of carbon fixation. They do so by coordinating CO_2_ assimilation rate, via buffering the bicarbonate pool, with generation of reducing power and energy currency and with oxygen utilization. This mechanism underlines the importance of the strategy for improving photosynthesis via engineering of bicarbonate pumps and increasing their efficient coupling with the reaction of Rubisco.

### Conflict of Interest Statement

The author declares that the research was conducted in the absence of any commercial or financial relationships that could be construed as a potential conflict of interest.

## Abbreviations

CA: carbonic anhydrase
MM: Michaelis–Menten
PGA: phosphoglyceric acid
RuBP: ribulose-1,5-bisphosphate
